# On the Relation of Cholera to Malarial Fever

**Published:** 1849-07

**Authors:** Charles W. Bell

**Affiliations:** Manchester


### PATHOLOGY AND PRACTICE OF MEDICINE.

*On the Relation of Cholera to Malarial Fever.* By Dr. CHARLES W. BELL, Manchester.—The circumstances under which Dr. Bell observed this disease in this country and in Persia, have been such as to impress him with the idea, that it is a variety of the malarial fevers, presenting the worst or (so called) *congestive* type of these diseases, in which the cold stage is prolonged and violent, and becomes in itself a source of danger. He describes his experience of cholera as follows:—

"After becoming acquainted with cholera, under very favorable circumstances, in Edinburgh in 1832–3, and in London in 1833–4, it was my lot to be stationed for several years in Persia, a country situated, both geographically and in point of climate, midway between India and Europe, and there I had the opportunity of observing closely the first approaches of the identical cholera which is now sweeping irresistibly towards us. The disease was there ushered in by a regular succession of epidemics, commencing in a fever apparently continued, but by and by assuming more the character of a remittent, and this very gradually changed to an intermittent or quotidian type; of this the cold stage gradually became prolonged, and assumed all the appearances of an attack of cholera; and then came the cholera, as it has everywhere been known, without any *obvious* stages or intermission. This again in its turn disappeared, and the epidemic resumed the character of remittent and continued fever for a time. These various changes occupied a period of eighteen months."

Dr. Bell thinks he has observed a remission in the collapse of cholera itself, about the eighteenth or twentieth hour from the commencement; but he does not lay much stress on this observation. The evacuations from the bowels he considers as the most usual and natural relief to that capillary congestion which is believed to be present both in this disorder and in intermittent fever. The consecutive fever of cholera is, according to the author, properly a remittent or quotidian type, which, when the disease exceeds the limit of three days, supplants the congestive form.

In this attempt to establish a relation between cholera and the quotidian intermittents, the author admits the difficulty which arises from the slight tendency displayed by cholera to continuance or recurrence. He thinks, however, that he has observed in other varieties of quotidian fevers a similar tendency to run a course of exactly three days; and indeed, from his own observation, seems disposed to regard this as a general law of quotidian intermittents.

Dr. Bell thinks that most of the epidemics of cholera will be found to have been ushered in by such a course of fevers as he has described in regard to Persia; and likewise to have been attended by an epidemic constitution, in which most other diseases tend to assume a congestive type. He has been enabled to trace the progress from personal observation of it in several places between Persia and this country, and has found it to be preceded and accompanied by these characteristic forms of disease. In particular, he regards the *relapsing fever* of this country, which was first accurately observed in Edinburgh in 1843, and has ever since prevailed in various localities, as a modification of one of the forms of *precursory* epidemic observed by him in Persia.

"In 1841, a fever of remittent and quotidian intermittent type broke out in Scinde, where it destroyed many of our best troops, alternating occasionally with cholera. Both proved severe in Caratchee; it spread through Beloochistan, and appeared at Bunder Abbas in the Persian Gulf early in 1842; also at Yezd. It thence spread westward to Shiraz, and northwards towards Ispahan and Tehran, proving every where extremely fatal; but its further progress to the northwest was arrested for a time, on the high grounds of Sultanieh, by the setting in of winter. Next spring it resumed its course, overspread Aderbijan, Erivan, Georgia, and the whole shores of the Caspian, crossed the Caucasus and was very fatal in Veronish in the centre of Southern Russia. I here lost sight of it in November 1843, but was not a little surprised to find it again in December, on my arrival in Edinburgh. It was there modified, it is true, and displayed less of an intermittent character, but was fully characterized by other symptoms, especially the tendency to relapse, and the pale tongue. Late in 1844, it appeared in Manchester, especially in Ancoats, where it was very severe. In 1845, it became epidemic in Liverpool, somewhat more modified in type, and the fever of 1846-7 in Manchester still preserved much of its peculiarities, especially in the frequency with which it was accompanied with jaundice, and in running a course of seven, fourteen or twenty-one days, and relapsing at these intervals."

The Persian epidemics were exceedingly varied in character, so much so that it appears scarcely possible to recognise some of them as fevers at all in any ordinary sense of the word. The disease appeared as tic doloureux, or intermitting hemicrania; paralysis, general or partial; apoplectic and epileptic symptoms; shivering ague, or ague without shivering.

"By and by this form was more frequently accompanied with vomiting, purging, and cramps, so as to constitute, in every respect, an intermitting cholera; whereas others had attacks resembling cholera in every particular, except that, instead of the more usual exudation from the bowels, this took place by the abundant outpouring of the fluid of the blood from the skin, or sometimes into the cellular texture, either of whole or part of the body, producing either partial or general dropsy in the course of a few hours. This latter form was more especially frequent in infants and children. Sometimes, too, this serous exudation occurred not less rapidly fatal than the worst kind of cholera, by producing suffocation, in consequence of sudden œdema of the lungs. All these, however, and various other anomalous affections, at length gave place to cholera in its ordinary form, with vomiting, purging, and spasms, as little marked by intermission as it ever is, and differing in no respect from that I had witnessed in Edinburg and London. Its temporary disappearance was then followed by the return of the intermittent fever."

The treatment suggested by Dr. Bell for cholera, as well as the whole of the affections to which he believes it to be allied, is the combination of quinine and iron, which he has found remarkably useful, not only as an anti-periodic, but in obviating congestion of the internal organs. He thinks bloodletting to be only useful when the disorder has not fairly commenced, and injurious when performed at a later period. —*Prov. Med. Journ.*

[The details of Dr. Bell's experience of individual cases in the original papers are most curious and interesting; and we strongly recommend their perusal to those interested in this subject as the fruit of a large experience in cholera, in a region little known, in a medical point of view to most Europeans. We think, however, that the author has decidedly overstepped the limits of sound induction in connecting together in one class diseases differing so much from each other as those he describes. The number of disorders which are admitted as manifestations of the precursory epidemic fevers is so great, and their kind so various, that we scarcely think it possible that cholera could at any time have invaded Persia without being heralded by some of them; and we cannot help thinking that the author has been betrayed into the recognition of analogies far to vague and indefinite when he associates apoplectic, paralytic, neuralgic, and dropsical attacks with ague, and all of these with cholera.

Suspending our judgment, however, as to the Persian epidemics, we must say, with more confidence, that we are convinced of the fallacy of Dr. Bell's ideas as to the relapsing fever of this country. The epidemic of Edinburgh in 1843, presented no resemblance whatever to a fever of "remittent or quotidian intermittent type," such as the author seems to have seen in Persia; nor did it resemble a malarious fever at all, except in the single circumstance of periodical relapse; and even here there was a marked difference, seeing that the relapse was not in the slightest degree influenced by the administration of quinine, as ascertained by the numerous trials in Edinburgh—(see Dr. Robertson's paper in *Monthly Journal* for November, 1848.) The origin of this fever by contagion was, we think, placed beyond all doubt by the researches of Dr. Henderson and others—(*Edin. Med. and Surg. Journal*, 1844;) and its connexion with endemic causes or with malaria cannot be said to be established or even rendered probable.

Neither can we admit that the relapsing fever has at any period of its history shown the slightest tendency to pass into the form of cholera. We think it satisfactorily proved that it has existed in Ireland during the last half century at least—(see Dr. Robert Paterson's article in *Edin. Med. and Surg. Journal*, Oct. 1848, or *Monthly Retrospect*, Nov. 1848); and during this period it has made several visits to this country, not one of which has been immediately followed by cholera. The epidemic of 1843 presented a remarkably fixed and definite character; it subsided without a trace of choleroid affection. It reappeared in Edinburgh, with nearly the same characters, in 1846–7, reached its acme without variation, and again subsided, without having ever suggested to any one, so far as we are aware, the idea of its being either cholera or quotidian intermittent. Finally, when cholera broke out in October 1848, the relapsing fever could not be said to exist in an epidemic form, and such cases of it as did occur in no way varied from the ordinary type. We think, therefore, that any supposed analogy of this fever, with intermittent on the one hand, or cholera on the other, is, so far as this country is concerned, entirely unwarranted by observation.—W. T. G.]

